# Molecular identification of trypanosomes in cattle in Malawi using PCR methods and nanopore sequencing: epidemiological implications for the control of human and animal trypanosomiases

**DOI:** 10.1051/parasite/2020043

**Published:** 2020-07-20

**Authors:** Megasari Marsela, Kyoko Hayashida, Ryo Nakao, Elisha Chatanga, Alex Kiarie Gaithuma, Kawai Naoko, Janelisa Musaya, Chihiro Sugimoto, Junya Yamagishi

**Affiliations:** 1 Division of Collaboration and Education, Research Center for Zoonosis Control, Hokkaido University Kita-20, Nishi-10, Kita-ku Sapporo 001-0020 Hokkaido Japan; 2 Laboratory of Parasitology, Veterinary Medicine Faculty, Hokkaido University Kita-18, Nishi-9, Kita-ku Sapporo 060-0818 Hokkaido Japan; 3 Department of Pathology, College of Medicine, University of Malawi P/Bag 360 Chichiri 30096 Blantyre 3 Malawi; 4 International Collaboration Unit, Research Center for Zoonosis Control, Hokkaido University Kita-20, Nishi-10, Kita-ku Sapporo 001-0020 Hokkaido Japan

**Keywords:** Cattle, Epidemiology, AAT, HAT, Malawi, Trypanosome

## Abstract

This study aimed to identify trypanosomes infecting cattle in Malawi in order to understand the importance of cattle in the transmission dynamics of Human African Trypanosomiasis (HAT) and Animal African Trypanosomosis (AAT). A total of 446 DNA samples from cattle blood from three regions of Malawi were screened for African trypanosomes by ITS1 PCR. The obtained amplicons were sequenced using a portable next-generation sequencer, MinION, for validation. Comparison of the results from ITS1 PCR and MinION sequencing showed that combining the two methods provided more accurate species identification than ITS1 PCR alone. Further PCR screening targeting the serum resistance-associated (SRA) gene was conducted to detect *Trypanosoma brucei rhodesiense. Trypanosoma congolense* was the most prevalent *Trypanosoma* sp., which was found in Nkhotakota (10.8%; 20 of 185), followed by Kasungu (2.5%; 5 of 199). Of note, the prevalence of *T. b. rhodesiense* detected by SRA PCR was high in Kasungu and Nkhotakota showing 9.5% (19 of 199) and 2.7% (5 of 185), respectively. We report the presence of animal African trypanosomes and *T*. *b*. *rhodesiense* from cattle at the human–livestock–wildlife interface for the first time in Malawi. Our results confirmed that animal trypanosomes are important causes of anemia in cattle and that cattle are potential reservoirs for human African trypanosomiasis in Malawi.

## Abstract (fr)

Cette étude visait à identifier les trypanosomes infectant les bovins au Malawi afin de comprendre l’importance des bovins dans la dynamique de transmission de la trypanosomiase humaine africaine (THA) et de la trypanosomose animale africaine (TAA). Au total, 446 échantillons d’ADN de sang de bovins provenant de trois régions du Malawi ont été soumis à un dépistage des trypanosomes africains par PCR de l’ITS1. Les amplicons obtenus ont été séquencés à l’aide d’un séquenceur portable de nouvelle génération, MinION, pour validation. La comparaison des résultats de la PCR de l’ITS1 et de la séquence MinION a montré que la combinaison des deux méthodes permettait une identification plus précise des espèces que la seule PCR de l’ITS1. Un autre dépistage par PCR ciblant le gène SRA (associé à la résistance du sérum) a été effectué pour détecter *Trypanosoma brucei rhodesiense*. *Trypanosoma congolense* était l’espèce de trypanosome la plus répandue, trouvée à Nkhotakota (10,8 % ; 20 sur 185), suivi de Kasungu (2,5 % ; 5 sur 199). Notamment, la prévalence de *T. b. rhodesiense* détectée par PCR de SRA était élevée à Kasungu et Nkhotakota, avec respectivement 9,5 % (19 sur 199) et 2,7 % (5 sur 185). Nous rapportons la présence de trypanosomes animaux africains et de *T. b. rhodesiense* de bovins à l’interface homme-bétail-faune sauvage, pour la première fois au Malawi. Nos résultats confirment que les trypanosomes animaux sont des causes importantes d’anémie chez les bovins et que les bovins sont des réservoirs potentiels pour la trypanosomiase humaine africaine au Malawi.

## Introduction

African trypanosomiasis is caused by protozoan parasites of *Trypanosoma* spp. and is mainly transmitted by tsetse flies [52]. This disease is a major concern in sub-Saharan Africa, with detrimental effects on both human and animal health and causing significant losses to affected countries [52]. Animal African trypanosomiasis (AAT) is caused by *T*. *congolense*, *T. vivax*, and *T. brucei brucei* [50]. Infection in domestic animals is usually severe, unlike in wildlife, where it is usually nonpathogenic [13]. AAT affects domestic animals, including cattle, goats, sheep, and pigs, and its pathogenicity differs according to the host species [13, 50]. Clinical symptoms include fever, anemia, loss of weight and productivity, abortion, decreased fertility, edema, paralysis, and even death [6]. AAT remains a major threat to animal health and stock farming within the tsetse belt [13]. Unlike *T. congolense* and *T. brucei,* which are transmitted by tsetse flies, *T. vivax* can also be transmitted mechanically by other hematophagous flies; as a result, it has a broader geographical distribution [44].

Human African trypanosomiasis (HAT) or sleeping sickness occurs in two forms with different features, due to *T. b. gambiense* and *T. b. rhodesiense* infections, respectively [8]. Gambiense HAT caused by *T*. *b. gambiense* is an anthroponotic disease that depends primarily on human-to-human transmission; humans act as the main reservoir, while animal reservoirs play a minor role [17]. It is distributed mostly in western and central Africa, an area that currently has 98% of reported cases of HAT [64]. Gambiense HAT is a chronic infection, during which a person can be infected for a long period of time without demonstrating major clinical signs of the disease. Symptoms often appear at the late stage of the disease, when the central nervous system is already affected [7]. In contrast, rhodesiense HAT caused by *T. b. rhodesiense* is a zoonotic disease that affects mainly animals (wildlife and livestock); humans are considered accidental hosts [17]. It is found in 13 countries in eastern and southern Africa, representing under 2% of reported cases of HAT, and it causes an acute infection [64]. The disease is known to progress quickly and invades the central nervous system right after onset of symptoms, a few months or weeks after infection [7].

Control of rhodesiense HAT is challenging because animals act as reservoirs for disease transmission [17, 63]. Although they do not show any clinical symptoms, animal reservoirs harbor parasites, and tsetse flies can acquire the infection [7]. Necessary control measures face obstacles because animal infections are difficult to monitor, unlike in humans, where they can be easily tracked. Wildlife have long been known to be the major reservoir of *T*. *b*. *rhodesiense* [41]. Livestock can also act as potent reservoir hosts for *T. b. rhodesiense* due to high exposure of humans to agriculture [61]. In Uganda, cattle were implicated as the principal domestic reservoirs of *T*. *b*. *rhodesiense* [60, 62], and they were also documented as reservoirs of *T*. *b*. *rhodesiense* in Kenya [59] and Tanzania [25].

In Malawi, rhodesiense HAT has been a burden for decades [18]. Unlike typical *T*. *b*. *rhodesiense* infections, HAT in Malawi is characterized by the distinct clinical sign of chronic hemolymphatic stage infection without the formation of a chancre; this makes diagnosis difficult [12, 29]. Endemic foci of HAT in the country are the Nkhotakota, Kasungu, and Rumphi districts [11, 30], where large national parks exist. Cattle are one of the most economically important livestock animals in Malawi [10, 48]. A previous study was conducted to update the distribution and clarify the epidemiology of bovine trypanosomiasis caused by *T*. *congolense*, *T*. *vivax*, and *T*. *brucei* in Malawi using an indirect AbELISA serological detection method [58]. However, the specificity of the IgG ELISA was questionable, and as in other serological assays, false positives may have been present [22, 58]. In contrast, PCR of the internal transcribed spacer (ITS) 1 region of ribosomal RNA (rRNA), which can distinguish species by product size, has been widely used to identify trypanosome species [19, 43]. Because the ITS1 region cannot resolve *Trypanozoon* species to the subspecies level (*T*. *brucei brucei*, *T*. *brucei rhodesiense*, *T*. *brucei gambiense*, *T*. *evansi*, and *T*. *equiperdum*) [19, 43], detection of *T. b. rhodesiense* by PCR has been widely conducted by targeting the human serum resistance-associated (SRA) gene [47]. Identifying species by ITS1 PCR is sometimes difficult due to ambiguous and nonspecific signals, which may result in false-positive annotations because of subjective human decisions. In addition, it is impossible to differentiate between *T. godfreyi* and *T. vivax* because they share overlapping amplicon size ranges.

MinION, developed by Oxford Nanopore Technologies, is a portable next-generation sequencer (NGS) that connects to a laptop computer through a USB cable [28, 36]. MinION is unique among sequencing tools because it identifies nucleotides in a nanoscale ion channel (nanopore) by detecting specific changes in the electric current when DNA passes through the nanopore [28]. DNA sequencing is a definitive diagnostic method for detecting pathogenic species, and several studies have reported application of MinION for pathogen identification [4, 21, 46, 53]. Unlike conventional sequencers, MinION is economically affordable, allowing sequence analysis without preinstallation of expensive equipment, and not requiring a separate electric supply after connecting to a laptop computer [53]. Further, operating MinION does not require sophisticated skills in biological research [53]. Given these features, genotyping of pathogens on-site with MinION is now feasible [53].

In this study, MinION NGS was combined to PCR-based methods to determine the prevalence of human and animal trypanosomes in cattle and to understand their epidemiological importance for HAT and AAT in Malawi.

## Materials and methods

### Ethics approval

The ethical clearance for animal sampling was obtained from the Ministry of Agriculture, Irrigation and Water Development in Malawi through the Department of Animal Health and Livestock Development with reference number 10/15/32/D.

### Sample collection

**Figure 1:**
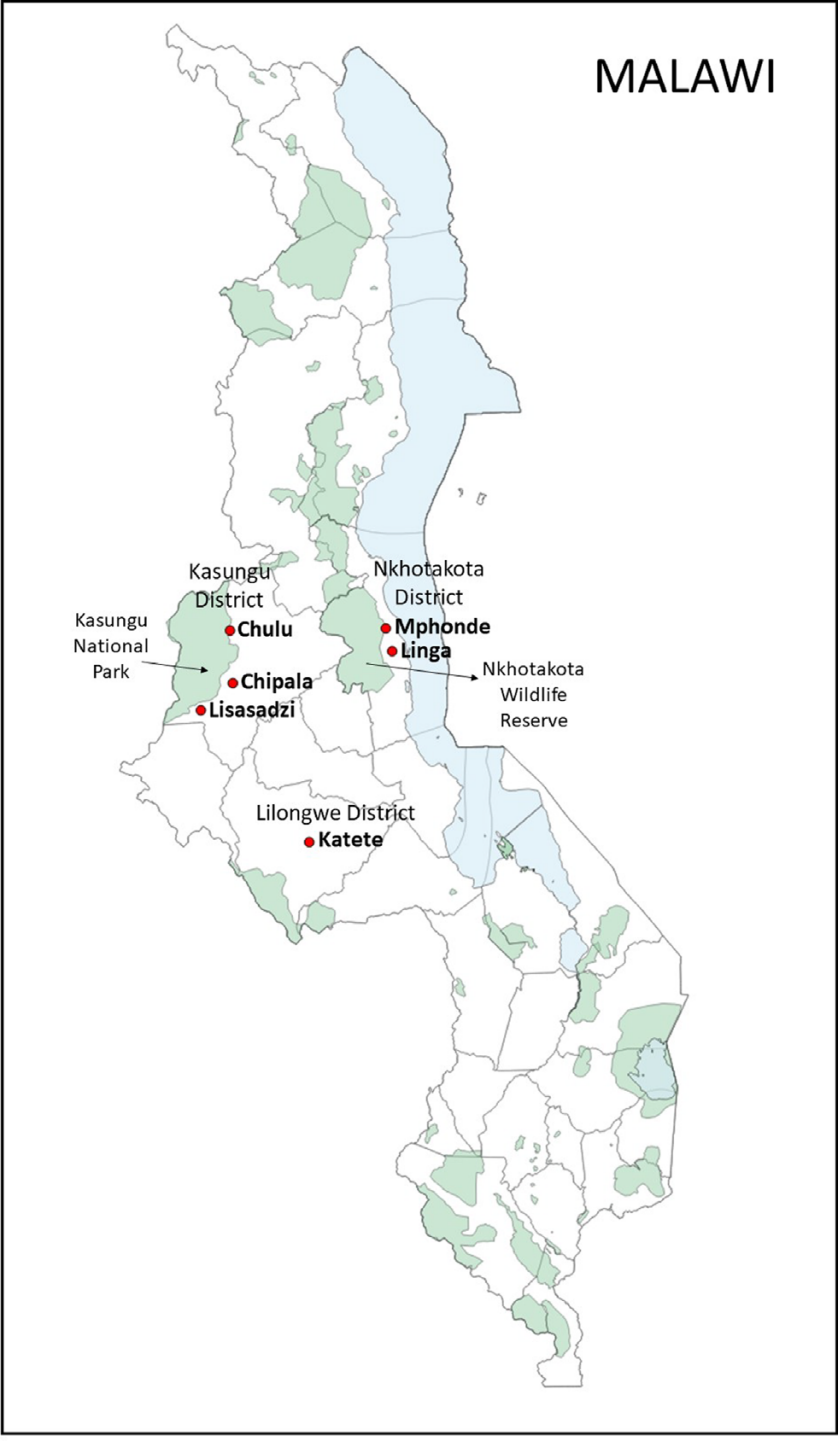
Map of Malawi showing sampling locations. Black arrows point to Kasungu National Park and Nkhotakota Wildlife Reserve. Sampling points in Nkhotakota and Kasungu districts were located outside the nature reserves in <1 km proximity.

This is a descriptive, cross-sectional study of trypanosome infection in cattle, conducted by analyzing DNA material from 446 bovine blood samples. Samples were collected from cattle in three districts in Malawi: Kasungu, Nkhotakota, and Lilongwe, in February and March 2018 during the rainy season. Information on farmers and cattle populations was obtained from the District Agriculture Development Office (DADO) for each district through their District Animal Health and Livestock Development Officer (DAHLDO). The sampling map (Fig. 1) was constructed using the free and open-source geographic information system QGIS [45].

Villages located in the vicinity of Kasungu National Park and Nkhotakota Wildlife Reserve were deliberately selected as sampling sites because these areas are locations of human–livestock–wildlife interface and are endemic for tsetse flies carrying *T. b. rhodesiense*. Both clinically healthy animals and animals with clinical signs were randomly selected within farms. In Kasungu and Nkhotakota districts, a total of 199 and 185 cattle blood samples were collected from 29 and 26 farms, respectively. These farms were selected on purpose from a farmer registry list (Supplementary Table 1), since the selected areas are situated in the human–livestock–wildlife interface area in the vicinity of Kasungu National Park and Nkhotakota Wildlife Reserve (Fig. 1). Both Kasungu National Park and Nkhotakota Wildlife Reserve are tsetse-infested areas and the major HAT/AAT foci in the country [11, 38, 58]. *Glossina morsitans morsitans* and *G. pallidipe*s are the dominant tsetse fly species in these areas, while *G. brevipalpis* is also known to exist to some extent [58]. The major cattle breed in these areas is Malawi Zebu, a local breed in the country also known as Angoni cattle (Small East African Zebu) in Eastern Zambia [39]. This breed is known to be susceptible to trypanosomiasis [57]. Cattle are kept in a free-range management system where animals are released during the day to graze freely and return home at dusk. Tsetse flies are often found at the sampling sites; thus, the frequencies of tsetse bite in the cattle in these areas are expected to be high. The other biting flies, tabanids, or *Stomoxys* spp. are also commonly found in these areas [9, 24].

In Lilongwe district, there are only two commercial farms, and 62 blood samples were collected from one of these farms. Holstein Friesian cattle are maintained with a semi-intensive farm management system, where animals are kept in paddocks. Tsetse flies are not found in this area, and no HAT/AAT cases have been reported in the past from this area [12, 58]. However, the other hematophagous flies including tabanids and *Stomoxys* spp. are commonly found in Lilongwe, which might facilitate the mechanical transmission of trypanosome, especially *T. vivax* [44].

Before sampling, the venipuncture site was disinfected with a methylated spirit swab. Then, 5 mL of blood were drawn by venipuncture of the external jugular vein into vacutainer EDTA tubes. Packed cell volume (PCV) counting was conducted to determine anemia; animals with a PCV *<* 24% were considered anemic. For molecular detection purposes, approximately 200 μL of blood were subjected to DNA extraction using a KURABO QuickGene DNA whole blood kit (Kurabo Industries Ltd.), following the manufacturer’s protocol.

### Detection of trypanosomes by ITS1 PCR

DNA samples were subjected to PCR amplification of the rRNA ITS1 region to identify all the pathogenic African trypanosome species/subspecies. ITS1 PCR was carried out using the primers described by Gaithuma et al. [19] (Supplementary Table 2). PCR was performed in a final volume of 25 μL, comprising 12.5 μL Ampdirect plus (Shimadzu, Japan), 0.125 μL BIOTAQ HS DNA Polymerase (5 U/μL) (Bioline, UK), 0.625 μL of each 10 mM primer, 0.5 μL of 2% DMSO, 8.625 μL RNase-free water, and 2 μL extracted DNA. The temperature and cycling profile included initial denaturation at 95 °C for 10 min followed by 37 cycles of denaturation at 94 °C for 30 s, annealing at 53 °C for 90 s, extension at 72 °C for 2 min, and final extension at 72 °C for 7 min. PCR products were examined by electrophoresis in 1.5% agarose S (Nippongene, Japan) in Tris-acetate EDTA (TAE) buffer and stained using GelRed (Biotium, USA) dye before being visualized under ultraviolet light in a transilluminator.

Signals on gel were judged by three investigators independently. They were classified as follows: positive (clear band), negative, and ambiguous (faint band or smearing). Trypanosome species were identified based on the size differences for members of the subgenus *Trypanozoon* (*T. b. brucei*, *T. evansi*, *T. b. rhodesiense*, and *T. b. gambiense*), a constant product of approximately 415–431 bp; for *T. congolense*, 560–705 bp; for *T. simiae*, 331–343 bp; for *T. godfreyi*, 220 bp; for *T. theileri*, 269–350 bp; and for *T. vivax*, 226–238 bp [19]. The plasmids that contain the TA-cloned ITS1 fragment of *T. congolense*, *T. brucei*, *T. vivax*, *T. godfreyi*, and *T. simiae* [40] were combined and used as a positive control for PCR and gel analysis.

### MinION library preparation for sequencing

To prepare a MinION sequencing library, amplicons of the ITS1 PCR were used as template then indexes were added by additional PCR. The indexed amplicons were further ligated with adapter DNA provided by Oxford nanopore; then, a sequence-ready library was obtained. Positive, ambiguous samples, and a positive control from ITS1 PCR were sequenced as follows. For the indexing, reagents comprised 5 μL Ampdirect plus (Shimadzu, Japan), 0.05 μL BIOTAQ HS DNA Polymerase (5 U/μL) (Bioline, UK), 0.2 μL of each 10 mM ITS1-index primers (Supplementary Table 2), 0.5 μL DMSO 2%, 2.55 μL RNase-free water, and 2 μL extracted DNA. PCR conditions were as follows: an initial hold at 95 °C for 10 min, followed by 10 cycles of 94 °C for 30 s, 60 °C for 1 min, 72 °C for 2 min, and a final extension at 72 °C for 5 min. Amplicon of the index PCR was prepared for MinION-compatible DNA libraries using a Ligation Sequencing Kit 1D SQK-LSK109 and Native Barcoding Kit EXP-NBD103 (Oxford Nanopore Technologies, UK) as per their instruction manuals. The indexed amplicons were purified by AMPure XP (Beckman Coulter, USA), and equal concentrations of each sample were pooled together to obtain 12 for each pool. End repair and dA-tailing were performed to the pooled, barcoded amplicons using the NEBNext UltraII End Repair/dA-Tailing module (New England Biolabs, UK) per the Oxford Nanopore 1D Native barcoding genomic DNA sequencing protocol. Subsequently, the purified, end-prepped DNA was ligated with the Adapter Mix (AMX) using the NEBNext Quick T4 DNA Ligase (New England Biolabs, UK). Final adapted DNA libraries were purified, and the pre-sequencing mix was eluted and quantified. The flowcell, FLO-MIN107 (Oxford Nanopore Technologies, UK), was primed for loading and sequencing of the library. Before sequencing, MinION and flowcell were assembled and connected to a computer. Platform QC was run using MinKNOW software.

### Detection of trypanosome species using MinION sequencing

Base-calling and de-barcoding were conducted using Guppy (Oxford Nanopore Technologies). De-indexing was performed using custom scripts. In brief, indexed primer sequences were aligned on each MinION read using LAST [26], and the best hit indexes at both terminals were subsequently assigned. De-multiplexed reads were aligned with the nucleotide dataset using BLASTn [3]. We kept the best hit for each read and counted the species name that appeared in the output, i.e., *T. vivax*, *T. godfreyi*, *T. evansi*, *T. brucei*, *T. equiperdum*, *T. congolense*, *T. simiae*, and *T. theileri*. The counts for *T*. *brucei*, *T*. *evansi*, and *T*. *equiperdum* were summed up and regarded together as *Trypanozoon*. Each taxon was programmatically assigned on the basis of the population of the read counts if it shared more than 20% of the total and more than 50 read counts. The prevalence of trypanosomes was determined according to the results of MinION sequencing. A schematic workflow of the MinION sequencing and bioinformatic analysis is illustrated in Supplementary Figure.

### Detection of *T. b. rhodesiense* by SRA PCR

SRA PCR was employed to identify *T*. *b*. *rhodesiense* using the primers described by Radwanska et al. [47] (Supplementary Table 2). Reagents used for each reaction included 5 μL Ampdirect plus (Shimadzu, Japan), 0.05 μL BIOTAQ HS DNA Polymerase (5 U/μL) (Bioline, UK), 0.2 μL of each 10 mM primer, 2.55 μL RNase-free water, and 2 μL extracted DNA. The temperature and cycling profile included an initial hold for 10 min at 95 °C, followed by 40 cycles at 94 °C for 30 s, 60 °C for 1 min, 72 °C for 1 min, and a final extension at 72 °C for 5 min. PCR products were examined by electrophoresis in 2% agarose S (Nippongene, Japan) in TAE buffer and stained using GelRed (Biotium, USA) dye before being visualized under ultraviolet light.

### Statistical analysis

The chi-square test was used to analyze the relationship between the infection rates by animals, region, and anemic status (*p* < 0.05).

## Results

### Trypanosome infections inferred from ITS1 PCR

**Figure 2:**
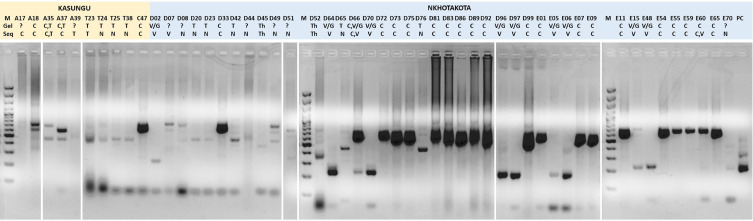
Gel images of the ITS1 PCR results for positive and ambiguous samples (*n* = 53, including one positive control). The combination of number and letter referred to the sample identity. M and PC represent the abbreviation of 1 kb marker and positive control, respectively. The letter below explains the interpretation of the gel (Gel) and the analysis of MinION sequencing (Seq). C, *T. congolense*; T, *Trypanozoon*; V/G, *T. vivax* or *T. godfreyi*; S, *T. simiae*; Th, *T. theileri*; N, no result; and “?”, ambiguous result.”

Forty-four positive, 8 ambiguous, and 394 negative samples were obtained by ITS1 PCR. For positive samples, we identified 21 amplicons with a single band size of 620–700 bp, indicating a single *T. congolense* infection; 9 amplicons with a single band size of 480 bp, indicating a single *Trypanozoon* infection; 9 amplicons with a single band size of 250 bp, indicating a single *T. vivax* or *T. godfreyi* infection; and 2 amplicons with a single band size of 350 bp, indicating a single *T. theileri* infection. Three amplicons showed clear multiple bands, indicating two mixed infections of *T. congolense* and *Trypanozoon* (A35 and A37; Fig. 2, Supplementary Table 3) and one mixed infection with *T. congolense* and *T. vivax* (D66; Fig. 2, Supplementary Table 3).

### Trypanosome infections inferred from MinION sequencing

In total, 503,039 reads were obtained from 53 multiplexed ITS1 amplicons, including one positive control. Taxonomic identity was determined for 369,673 reads using a BLAST homology search. For *T. congolense*, 24 samples (including single and mixed infections) were positive by ITS1 PCR, and *T. congolense* sequences were detected in the same 24 samples and one additional sample, A17, by the MinION system (Fig. 2, Supplementary Table 3). For *T. vivax*, 10 samples were positive, and the same 10 samples were confirmed by sequencing, as well as two additional samples, D07 and E60. E60 was a mixed *T. congolense* and *T. vivax* infection. For *T. theileri*, two samples were positive in both ITS1 PCR and the MinION system.

In contrast, 11 samples positive for *Trypanozoon* were identified by ITS1 PCR. However, using the MinION system, *Trypanozoon* sequences were detected in only 3 of these 11 products (Supplementary Table 3). Most cases, with the exception of A37, produced reads for *Trypanozoon* that were much lower than our threshold (50 reads minimum). For A37, 172 reads for *Trypanozoon* out of 2965 reads were obtained, but reads were below our criteria, with a minimum of 50 reads and more than 20% of total reads; therefore, this sample was negative even though we could not eliminate the possibility of actual *Trypanozoon* infection.

Three confirmed cases of mixed infections were observed (A35, D66, and E60; Fig. 2, Supplementary Table 3). E60 showed a clear signal for *Trypanozoon* and a faint signal for *T. godfreyi* or *T. vivax*. However, the MinION system provided substantial numbers of sequences to show that the sample was a mixed infection of *T. congolense* and *T. vivax* (Supplementary Table 3). Another case, A37, appeared to be a mixed infection of *T. congolense* and *Trypanozoon* by ITS1 PCR; however, MinION results did not support *Trypanozoon* infection as described above.

### Prevalence of African trypanosomes determined by ITS1-MinION and SRA-PCR

In addition to ITS1-MinION, SRA PCR was conducted to differentiate *T. b. rhodesiense* from *Trypanozoon*. The samples of ITS1-MinION positive for *Trypanozoon* and SRA-PCR negative were assigned as *T. b. brucei*. The samples of SRA-PCR positive and ITS1-MinION negative were assigned as *T. b. rhodesiense*, since SRA PCR has higher sensitivity as discussed later. *Trypanosoma congolense* was the most prevalent trypanosome detected in Nkhotakota (10.8%; 20 of 185) and Kasungu (2.5%; 5 of 199). *Trypanosoma vivax* was found in Nkhotakota (6.5%; 12 of 185). Non-pathogenic *Trypanosoma* species *T. theileri* (1.1%; 2 of 185) was also detected in Nkhotakota. There were three mixed infections: one *T. congolense* with *T. b. brucei*, and two *T. congolense* with *T. vivax* (Table 1).

**Table 1:** Detection of pathogenic trypanosome species by analysis of SRA PCR and MinION sequencing of ITS1 PCR products.

Sampling sites	Total number of samples	Number of positive samples				
		*T. congolense*	*T. vivax*	*Trypanozoon*		Any trypanosome species
				*T. b. brucei*	*T. b. rhodesiense*	
Kasungu	199	5 [1] (2.5%)	0 (0%)	2 [1] (0.4%)	19 (9.5%)	25 (12.6%)
Nkhotakota	185	20 [2] (10.8%)	12 [2] (6.5%)	0 (0%)	5 (2.7%)	35 (18.9%)*
Lilongwe	62	0 (0%)	0 (0%)	0 (0%)	0 (0%)	0 (0%)
Total	446	25	12	2	24	60
Prevalence		5.6%	2.7%	0.4%	5.4%	13.4%

Numbers in brackets [*n*] indicate samples with a coinfection involving multiple trypanosomes. Numbers in parentheses represent the prevalence of each trypanosome, or infected cattle per district. Asterisk (*) indicates that the prevalence of in Nkhotakota is significantly higher than in Lilongwe and Kasungu (*p* < 0.05). Samples with *T. theileri* are counted as negative in this table.

*Trypanosoma brucei rhodesiense* was detected in cattle in both HAT-active foci by SRA PCR. A higher prevalence of *T. b. rhodesiense* was found in samples from Kasungu (9.5%; 19 of 199), followed by Nkhotakota (2.7%; 5 of 185) (Table 1). The detected number of *T. b. rhodesiense* was much higher than the detected number of *Trypanozoon* as indicated by the ITS1-MinION detection system. Out of 24 SRA PCR-positive samples, only 1 sample (B23) was positive when analyzed by the ITS1-MinION detection system (Supplementary Table 3).

These trypanosome parasites were all detected in two districts, with the highest prevalence being in Nkhotakota (18.9%; 35 of 185), followed by Kasungu (12.6%; 25 of 199) (Table 1), while no parasites were detected in Lilongwe samples. There was a significant difference in prevalence between the study sites (*p* < 0.05).

### Correlation between infection by African trypanosomes and the anemic status of cattle

There was a significant difference in the anemic status (PCV < 24%) between cattle with trypanosome infection and without infection (*p* < 0.05) (Table 2). Of 16 anemic cattle, 11 heads (68.7%) were positive for either *T. congolense*, *T. vivax* or *Trypanozoon* (*T. b. brucei* and *T. b. rhodesiense*) infections. In contrast, of 430 non-anemic cattle, only 49 heads (11.4%) were positive for trypanosome infection. In particular, infection with *T. congolense* showed a significant difference in anemia status compared to other species (*p* < 0.05). A significant difference in the anemic status of *T. congolense*-infected cattle was observed in both single-infected cattle and mixed-infected cattle (Table 2).

**Table 2:** Associations between PCV values and trypanosome species infected in cattle.

PCV	Status	*T. congolense*	*T. vivax*	*T. b. brucei*	*T. b. rhodesiense*	All trypanosomes	Total animals	No trypanosomes
<24%	Anemic	62.5% (10/16)*	12.5% (2/16)	6.2% (1/16)	0% (0/16)	68.7% (11/16)*	16	5
24–50%	Normal	3.5% (15/430)	2.3% (10/430)	0.5% (1/430)	5.6% (24/430)	11.4% (49/430)	430	404
Total		5.6%	2.7%	0.4%	5.4%	13.4% (60/446)	446	409

The proportion in parentheses (*n*/*n*) represents the number of trypanosome-infected animals compared to the total number of animals. Asterisk (*) indicates that the anemic status in *T. congolense* infection is significantly higher than infection with *T. vivax* and *Trypanozoon* (*p* < 0.05).

## Discussion

In this study, we applied next-generation sequencing (NGS) technologies, particularly MinION, a field-friendly, portable, rapid, and affordable NGS device. ITS1 amplicons, including ambiguous signals, were subjected to sequencing analysis. Identification of *Trypanosoma* sp. by ITS1 PCR and sequencing analysis was largely consistent for *T. congolense*, *T. theileri*, and *T. vivax*. This suggests that MinION sequencing can detect and differentiate trypanosome species. In addition, MinION sequencing appreciably remedied four downsides of ITS1 PCR: ambiguous signals, similar amplicon sizes, nonspecific signals, and multiple infections. We successfully detected a substantial number of reads from samples with ambiguous ITS1 PCR amplicons. *T. godfreyi* and *T. vivax* were differentiated. On the other hand, nonspecific amplification hampers correct identification and can lead to false positives. We observed 11 signals of approximately 450 bp corresponding to *Trypanozoon*; however, most were not supported by sequencing. These could be annotated as false positives without sequencing validation. Apart from nonspecific signals, all samples definitively annotated by the ITS1 PCR had more sequence reads than the threshold of 50. Since we detected enough reads for other trypanosome species from ITS1 PCR, the discrepancy presumably derived from nonspecific PCR amplification of fragments of the same size as the *Trypanozoon* amplicon. Thus, in several cases, disagreement between ITS1-PCR gel analysis and ITS1-MinION sequence analysis was observed, likely due to nonspecific amplification and misjudgement of the ITS1-PCR gel analysis. Multiple infections were also successfully confirmed by sequencing. All these observations support the assertion that ITS1 PCR validated by sequencing analysis using MinION increases the reliability of parasite detection. Conversely, we obtained only tentative annotation in some samples, even though substantial sequence reads were acquired. Annotation depends on the threshold, and we applied a conservative value to eliminate false positives rather than allow false negatives. BLAST homology search and de-indexing steps were possible sources of error owing to the low accuracy of MinION sequencing. The effective range of ratios and absolute numbers of parasites in mixed infections should also be determined. These points should be clarified and optimized in future studies. The combined approach of ITS1 PCR and MinION has the potential to exclude false positives by nonspecific amplification and less-objective human decisions. Sequencing itself became cost-effective because of the multiplex sequencing system using MinION indexes and barcodes. The quality of field samples is occasionally poor and leads to unexpected nonspecific amplification, as observed in our cases. MinION provides sequence information that can exclude nonspecific amplification and thus improve specificity [65]. Therefore, utilization of MinION-based sequencing in this study will help increase the reliability of PCR-based epidemiology.

ITS1 PCR in combination with MinION sequencing provides sequence information to identify a broad range of trypanosome species in a more reliable manner compared to the conventional ITS1 PCR with gel analysis only [19]. However, this method has a limitation in characterizing *Trypanozoon* subspecies owing to the highly conserved sequence of the species [14]. In addition, ITS1 PCR is known to have less sensitivity compared to other published primer sets targeting repeat sequences [1, 34, 49], or SRA PCR [51]. In our study, most of the SRA-PCR-positive samples were negative for ITS1 PCR, suggesting that ITS1 PCR alone is of low sensitivity and cannot used to investigate the prevalence of *Trypanozoon* especially for *T. b. rhodesiense* as reported before [49]. However, as species-specific primers cannot identify other trypanosomes, the ITS1 PCR system offers value for detecting the parasites broadly in the same reaction with less time and cost. To make the ITS1-MinION system more useful for epidemiological studies, future studies should be required to develop new primers targeting ITS1 or other regions, as a simultaneous sensitive diagnosis method. In the current ITS1-MinION system, the SRA PCR test is still recommended for screening human-infective *T. b. rhodesiense* in a more sensitive and specific manner.

In this study, the most common trypanosome detected was *T*. *congolense*. This is in agreement with reports from other southern African countries [32, 58]. *Trypanosoma congolense* and *T. vivax* were more prevalent in Nkhotakota than in Kasungu, with no parasites seen in Lilongwe. Possible reasons for this trend may be the different methods applied for AAT control. Nkhotakota wildlife services have intensified blue cloth targets in the park to reduce the number of tsetse flies. In Kasungu, local veterinarians and farmers apply an anti-parasitic treatment, Berenil (diminazene aceturate), to the cattle twice every year before and after the herding season. Berenil is a common therapeutic and preventive medicine for animal trypanosomiasis that has been used for >60 years [27]. Since samples were collected during the rainy season, we may expect a higher prevalence of parasites in the dry season when disease transmission peaks [31, 42]. In both the Kasungu and Nkhotakota areas, cattle are usually bitten when brought into close contact with flies at river crossings, village water holes, or other tsetse fly habitats in the field [37]. On the other hand, semi-intensive farming was applied in Lilongwe, where the cattle were kept in paddocks, thus preventing the mechanical transmission of *T*. *vivax* in cattle by biting flies. However, as *T. vivax* was detected in Nkhotakota, this emphasizes the necessity of sustained non-tsetse vector control for animal trypanosomiasis in the region. Also, since we collected a limited number of cattle samples only at three locations during short period, a broader countrywide survey is required to assess the overall AAT/HAT situation in the country.

The PCV value of infected cattle was significantly lower than that of non-infected cattle. Anemia has long been considered the main clinical sign of trypanosomiasis in both humans and animals [15, 16]. Anemia associated with infection has been previously associated with lower productivity of infected animals [55, 56]. Consistent with previous studies [33, 54], our findings confirmed that cattle with *T. congolense* infection tended to be anemic, as compared to those infected with other trypanosome species, suggesting the importance of controlling AAT.

Malawi is rich in ecosystems where humans, livestock, and wildlife populations exist close to each other. Compared with neighboring countries such as Uganda, Kenya, and Tanzania in eastern and southern Africa where rhodesiense HAT is endemic [5, 59, 62], *T*. *b*. *rhodesiense* prevalence in tsetse flies is higher in Malawi, according to xenomonitoring data [2]. As Kasungu and Nkhotakota are hotspots of HAT in Malawi, the presence of *T. b. rhodesiense* in cattle has been suspected in these areas [11, 38], but had not been investigated before this study.

Studying the human–livestock–wildlife interface is essential now, and even more so in the future because of human population expansion and agricultural development [20]. This expansion induces farmers and their livestock to migrate closer to wildlife conservation areas, increasing their exposure to tsetse flies. Here, the surveyed areas were in close proximity, within <1 km, to the Kasungu and Nkhotakota national parks. The extensive farming system allowed cattle to interact with infected wildlife and tsetse flies during grazing activities at the human–livestock–wildlife interface, increasing the possibility of pathogen sharing and disease transmission in the populations involved.

In this study, we identified both human- and animal-infective trypanosomes residing in cattle at the human–livestock–wildlife interface areas in Malawi. A limited number of trypanosomiasis and tsetse control programs addressing both diseases have been conducted in the area [35]. AAT and HAT control activities are interdependent since both diseases share the same transmission vector and host. Control programs targeting flies and animal populations are necessary to achieve HAT control [23]. This study contributes to improving knowledge regarding the status of trypanosomiasis in Malawi. Our findings emphasize the need for sustainable integration between AAT and HAT control measures and collaborative human and animal health care services under the One Health concept, which are indispensable in tackling HAT.

## Conclusions

This is the first study to assess the prevalence of animal and human infective trypanosomes in cattle in Malawi. The use of MinION sequencing in combination with ITS1 PCR increased the reliability of PCR-based comprehensive trypanosome detection. However, for more sensitive and specific detection of human infective *T. b. rhodesiense*, SRA PCR is still recommended. Trypanosome-susceptible cattle harbor both human and animal infective trypanosomes, implying its role as a potential reservoir of *T*. *b*. *rhodesiense*. This study emphasizes the urgent need for sustainable control measures within the context of the One Health approach for AAT and HAT in livestock.

## Supplementary materials

Supplementary material is available at https://www.parasite-journal.org/10.1051/parasite/2020043/olm
Supplementary Table 1 Farmer registry list used during blood samples collection in the districts of Kasungu and Nkhotakota. (*) indicates the data obtained from the District Agriculture Development Office (DADO) for each district through their District Animal Health and Livestock Development Officer (DAHLDO). Supplementary Table 2 List of primers and their sequences used in this study. Supplementary Table 3 SRA PCR, ITS PCR, and MinION sequencing analysis for characterizations of trypanosome species. No hit* shows the number of reads that could not be identified by BLAST. FASTA $ describes the total number of obtained reads processed into BLAST. “PC+” and “?” refer to positive control and ambiguous results, respectively. Samples which were negative in both the SRA PCR and the ITS1 PCR are not shown here. Supplementary Figure Workflow for molecular detection of African trypanosomes. The workflow depicts structural steps for detection of African animal and human trypanosomes with serial arrows to the right for cattle samples, and a single downward arrow for human samples. The section on the identification of trypanosome species by sequencing is divided into three steps: library preparation, sequencing and basecalling, and de-multiplexing. Bioinformatic analyses required in the experiments are framed in red borderline.
