# Supplementary material for: Molecular identification of trypanosomes in cattle in Malawi using PCR methods and nanopore sequencing: epidemiological implications for the control of human and animal trypanosomiases
Source: Parasite. 2020 Jul 20;27:46. doi: 10.1051/parasite/2020043 (PMC7370688; doi:10.1051/parasite/2020043)
Supplement: Supplementary Table 1 — Farmer registry list used during blood samples collection in the districts of Kasungu and Nkhotakota. (*) indicates the data obtained from the District Agriculture Development Office (DADO) for each district through their District Animal Health and Livestock Development Officer (DAHLDO). [file parasite-27-46-s1.pdf]

Supplementary Table 1. Farmer registry list used during blood samplescollection in the districts of Kasungu and Nkhotakota.

(*) indicates the data obtained from the District Agriculture Development Office (DADO) for each district through their District Animal Health and Livestock Development Officer (DAHLDO).

District	Subdistrict	Total number of farms*	Number of sampled farms	Number of sampled cattle
Kasungu	Chulu	47	15	62
	Lisasadzi	20	7	72
	Chipala	19	7	65
Nkhotakota	Mphonde	29	11	84
	Linga	43	15	101
