# Supplementary material for: Molecular identification of trypanosomes in cattle in Malawi using PCR methods and nanopore sequencing: epidemiological implications for the control of human and animal trypanosomiases
Source: Parasite. 2020 Jul 20;27:46. doi: 10.1051/parasite/2020043 (PMC7370688; doi:10.1051/parasite/2020043)
Supplement: Supplementary Table 2 — List of primers and their sequences used in this study. [file parasite-27-46-s2.pdf]

Supplementary Table 2. List of primers and their sequences used in this study.

Primer name	Primer sequence 5'- 3'
Forward AITS1	CGGAAGTTCACCGATATTGC
Reverse AITS1	AGGAAGCCAAGTCATCCATC
Forward AITS1-index	[index 1-12]-CGGAAGTTCACCGATATTGC
Reverse AITS1-index	[index 1-12]-AGGAAGCCAAGTCATCCATC
Index1	CTATACAGCATGAG
Index2	AGAGTCTAGCTAGC
Index3	TGCGACACATGTGA
Index4	GACTATGCAGTGCA
Index5	ACGCGTGCATCTAC
Index6	TCGAGTAGTCTCAG
Index7	GTATCATGTCAGCA
Index8	AGCTAGTAGCTACT
Index9	CGAGACGATACTCT
Index10	TAGATGCTCGCGAG
Index11	GCTACGCTGAGTAG
Index12	TCTCAGCGCAGTGA
Forward SRA	ATAGTGACAAGATGCGTACTCAACGC
Reverse SRA	AATGTGTTCGAGTACTTCGGTCACGCT
