# Supplementary material for: Molecular identification of trypanosomes in cattle in Malawi using PCR methods and nanopore sequencing: epidemiological implications for the control of human and animal trypanosomiases
Source: Parasite. 2020 Jul 20;27:46. doi: 10.1051/parasite/2020043 (PMC7370688; doi:10.1051/parasite/2020043)
Supplement: Supplementary Table 3 — SRA PCR, ITS PCR, and MinION sequencing analysis for characterizations of trypanosome species. No hit* shows the number of reads that could not be identified by BLAST. FASTA $ describes the total number of obtained reads processed into BLAST. “PC+” and “?” refer to positive control and ambiguous results, respectively. Samples which were negative in both the SRA PCR and the ITS1 PCR are not shown here. [file parasite-27-46-s3.pdf]

Supplementary Table 3. SRA PCR, ITS PCR, and MinION sequencing analysis for characterizations of trypanosome species.

No hit* shows the number of reads that could not be identified by BLAST. FASTA $ describes the total number of obtained reads processed into BLAST. “PC+” and “?” refer to positive control and ambiguous results, respectively.

Samples which were negative in both the SRA PCR and the ITS1 PCR are not shown here.

No.	Sample ID	Region	SRA PCR	ITS1 MinION		Species determined in this study	MinION analysis										
				Gel image result of PCR	MinION sequencing analysis		Number of hit reads by BLAST									No hit*	Fasta $
							T. vivax	*T. godfreyi*	*Trypanozoon*	*T. congolense*	*T. simiae*	*T. theileri*	*Trypanosoma sp.*	Others	Total		
1	A17	Kasungu		?	*T. congolense*	*T. congolense*	9	0	519	5605	0	25	346	1774	8278	3930	12208
2	A18	Kasungu		*T. congolense*	*T. congolense*	*T. congolense*	0	1	1	1188	0	54	17	160	1421	1053	2474
3	A20	Kasungu	*T. b. rhodesiense*			*T. b. rhodesiense*											
4	A35	Kasungu		*T. congolense, Trypanozoon*	*T. congolense, Trypanozoon*	*T. congolense, T. b. brucei*	1	0	353	905	0	0	28	18	1305	216	1521
5	A37	Kasungu		*T. congolense, Trypanozoon*	*T. congolense*	*T. congolense*	1	1	172	2273	3	0	11	7	2468	497	2965
6	A39	Kasungu		?	*Trypanozoon*	*T. b. brucei*	3	0	1209	5	0	0	172	52	1441	1432	2873
7	A46	Kasungu	*T. b. rhodesiense*			*T. b. rhodesiense*											
8	A51	Kasungu	*T. b. rhodesiense*			*T. b. rhodesiense*											
9	A53	Kasungu	*T. b. rhodesiense*			*T. b. rhodesiense*											
10	A54	Kasungu	*T. b. rhodesiense*			*T. b. rhodesiense*											
11	B04	Kasungu	*T. b. rhodesiense*			*T. b. rhodesiense*											
12	B06	Kasungu	*T. b. rhodesiense*			*T. b. rhodesiense*											
13	B08	Kasungu	*T. b. rhodesiense*			*T. b. rhodesiense*											
14	B13	Kasungu	*T. b. rhodesiense*			*T. b. rhodesiense*											
15	B14	Kasungu	*T. b. rhodesiense*			*T. b. rhodesiense*											
16	B17	Kasungu	*T. b. rhodesiense*			*T. b. rhodesiense*											
17	B19	Kasungu	*T. b. rhodesiense*			*T. b. rhodesiense*											
18	B23	Kasungu	*T. b. rhodesiense*	*Trypanozoon*	*Trypanozoon*	*T. b. rhodesiense*	4	0	278	2	0	23	24	5	336	552	888
19	B24	Kasungu		*Trypanozoon*	Negative		1	0	0	6	0	0	0	58	65	451	516
20	B25	Kasungu		*Trypanozoon*	Negative		4	0	16	2	0	24	6	17	69	361	430
21	B38	Kasungu		*Trypanozoon*	Negative		111	0	21	2	0	0	61	32	227	1735	1962
22	B42	Kasungu	*T. b. rhodesiense*			*T. b. rhodesiense*											
23	B53	Kasungu	*T. b. rhodesiense*			*T. b. rhodesiense*											
24	B68	Kasungu	*T. b. rhodesiense*			*T. b. rhodesiense*											
25	C21	Kasungu	*T. b. rhodesiense*			*T. b. rhodesiense*											
26	C35	Kasungu	*T. b. rhodesiense*			*T. b. rhodesiense*											
27	C47	Kasungu		*T. congolense*	*T. congolense*	*T. congolense*	59	327	0	1953	0	0	280	12	2631	971	3602
28	C50	Kasungu	*T. b. rhodesiense*			*T. b. rhodesiense*											
29	D02	Nkhotakota		*T. vivax* or *T.godfreyi*	*T. vivax*	*T. vivax*	4676	0	0	7	0	0	346	1	5030	3154	8184
30	D07	Nkhotakota		?	*T. vivax*	*T. vivax*	66	1	3	6	0	0	4	41	121	149	270
31	D08	Nkhotakota		?	Negative		13	0	1	8	0	0	0	197	219	2232	2451
32	D11	Nkhotakota	*T. b. rhodesiense*			*T. b. rhodesiense*											
33	D15	Nkhotakota	*T. b. rhodesiense*			*T. b. rhodesiense*											
34	D20	Nkhotakota		*Trypanozoon*	Negative		0	0	0	19	0	0	0	111	130	2210	2340
35	D23	Nkhotakota		*Trypanozoon*	Negative		1	0	0	26	0	1	0	26	54	447	501
36	D33	Nkhotakota		*T. congolense*	*T. congolense*	*T. congolense*	0	0	0	4167	0	0	6	2	4175	240	4415
37	D37	Nkhotakota	*T. b. rhodesiense*														
38	D42	Nkhotakota		*Trypanozoon*	Negative		1	0	0	30	0	7	0	7	45	501	546
39	D44	Nkhotakota		?	Negative		5	3	0	5	4	22	8	20	67	748	815
40	D45	Nkhotakota		*T. theileri*	*T. theileri*	*T. theileri*	58	0	0	2	0	457	52	159	728	448	1176
41	D49	Nkhotakota		?	Negative		162	0	0	14	0	47	26	981	1230	4515	5745
42	D51	Nkhotakota		?	Negative		61	1	0	4	0	11	11	2064	2152	1433	3585
43	D52	Nkhotakota		*T. theileri*	*T. theileri*	*T. theileri*	194	0	0	0	0	517	64	42	817	286	1103
44	D64	Nkhotakota		*T. vivax* or *T.godfreyi*	*T. vivax*	*T. vivax*	19991	10	2	14	11	3	2518	17	22566	16442	39008
45	D65	Nkhotakota		*Trypanozoon*	Negative		19	0	0	21	0	7	3	139	189	1197	1386
46	D66	Nkhotakota		*T. congolense* ,<br>*T. vivax* or *T. godfreyi*	*T. congolense* , *T. vivax*	*T. congolense* , *T. vivax*	6096	0	0	7422	0	0	332	2	13852	6010	19862
47	D70	Nkhotakota		*T. vivax* or *T.godfreyi*	*T. vivax*	*T. vivax*	1665	0	0	13	0	0	52	1	1731	1051	2782
48	D72	Nkhotakota		*T. congolense*	*T. congolense*	*T. congolense*	55	0	0	6752	0	0	12	19	6838	661	7499
49	D73	Nkhotakota		*T. congolense*	*T. congolense*	*T. congolense*	264	0	0	2242	3	0	56	5	2570	1451	4021
50	D75	Nkhotakota		*T. congolense*	*T. congolense*	*T. congolense*	4	0	0	21190	0	1	31	27	21253	2409	23662
51	D76	Nkhotakota		*Trypanozoon*	Negative		1	0	0	385	0	0	0	540	926	9635	10561

52	D81	Nkhotakota		*T. congolense*	*T. congolense*	*T. congolense*	0	0	0	49935	0	0	52	9	49996	4983	54979
53	D83	Nkhotakota		*T. congolense*	*T. congolense*	*T. congolense*	0	0	0	5607	0	0	8	3	5618	557	6175
54	D86	Nkhotakota		*T. congolense*	*T. congolense*	*T. congolense*	6	0	0	15852	0	0	16	25	15899	1448	17347
55	D89	Nkhotakota		*T. congolense*	*T. congolense*	*T. congolense*	9	5	2	2818	4	0	100	28	2966	1273	4239
56	D92	Nkhotakota		*T. congolense*	*T. congolense*	*T. congolense*	130	9	2	806	12	0	19	2	980	265	1245
57	D96	Nkhotakota		*T. vivax* or *T.godfreyi*	*T. vivax*	*T. vivax*	586	6	2	9	10	0	27	3	643	653	1296
58	D97	Nkhotakota		*T. vivax* or *T.godfreyi*	*T. vivax*	*T. vivax*	3183	0	0	11	0	0	187	5	3386	5869	9255
59	D99	Nkhotakota		*T. congolense*	*T. congolense*	*T. congolense*	27	0	0	12565	0	0	13	4	12609	1384	13993
60	E01	Nkhotakota		*T. congolense*	*T. congolense*	*T. congolense*	10	0	0	10592	0	0	9	7	10618	1119	11737
61	E05	Nkhotakota		*T. vivax* or *T.godfreyi*	*T. vivax*	*T. vivax*	3681	1	0	36	0	0	238	5	3961	1740	5701
62	E06	Nkhotakota		*T. vivax* or *T.godfreyi*	*T. vivax*	*T. vivax*	2922	0	0	23	25	0	157	2	3129	2868	5997
63	E07	Nkhotakota		*T. congolense*	*T. congolense*	*T. congolense*	20	0	0	9592	0	9	16	7	9644	837	10481
64	E09	Nkhotakota		*T. congolense*	*T. congolense*	*T. congolense*	4	0	0	14794	0	0	7	127	14932	1214	16146
65	E11	Nkhotakota		*T. congolense*	*T. congolense*	*T. congolense*	3	0	0	12758	0	0	13	7	12781	1116	13897
66	E15	Nkhotakota		*T. vivax* or *T.godfreyi*	*T. vivax*	*T. vivax*	14753	0	0	57	0	58	595	682	16145	13594	29739
67	E29	Nkhotakota	*T. b. rhodesiense*			*T. b. rhodesiense*											
68	E32	Nkhotakota	*T. b. rhodesiense*			*T. b. rhodesiense*											
69	E48	Nkhotakota		*T. vivax* or *T.godfreyi*	*T. vivax*	*T. vivax*	3061	0	0	121	0	1	180	7	3370	1380	4750
70	E54	Nkhotakota		*T. congolense*	*T. congolense*	*T. congolense*	57	0	0	10470	0	0	9	46	10582	637	11219
71	E55	Nkhotakota		*T. congolense*	*T. congolense*	*T. congolense*	145	0	0	5908	0	15	20	31	6119	527	6646
72	E59	Nkhotakota		*T. congolense*	*T. congolense*	*T. congolense*	16	1	1	15840	0	28	27	37	15950	2372	18322
73	E60	Nkhotakota		*T. congolense*	*T. congolense, T. vivax*	*T. congolense, T. vivax*	2529	192	0	2051	11	0	285	3	5071	1720	6791
74	E65	Nkhotakota		*T. congolense*	*T. congolense*	*T. congolense*	18	0	0	31134	0	0	46	357	31555	3999	35554
75	E70	Nkhotakota		?	Negative		1907	130	0	323	63	0	1560	20	4003	6607	10610
76	PC+			*T. vivax* or *T.godfreyi*,<br>*T. simiae*, faint *Trypanozoon*	*T. vivax*		10318	4516	589	1610	5605	0	4144	0	26782	10787	37569
