# Supplementary figures and images for: Molecular identification of trypanosomes in cattle in Malawi using PCR methods and nanopore sequencing: epidemiological implications for the control of human and animal trypanosomiases
Source: Parasite. 2020 Jul 20;27:46. doi: 10.1051/parasite/2020043 (PMC7370688; doi:10.1051/parasite/2020043)

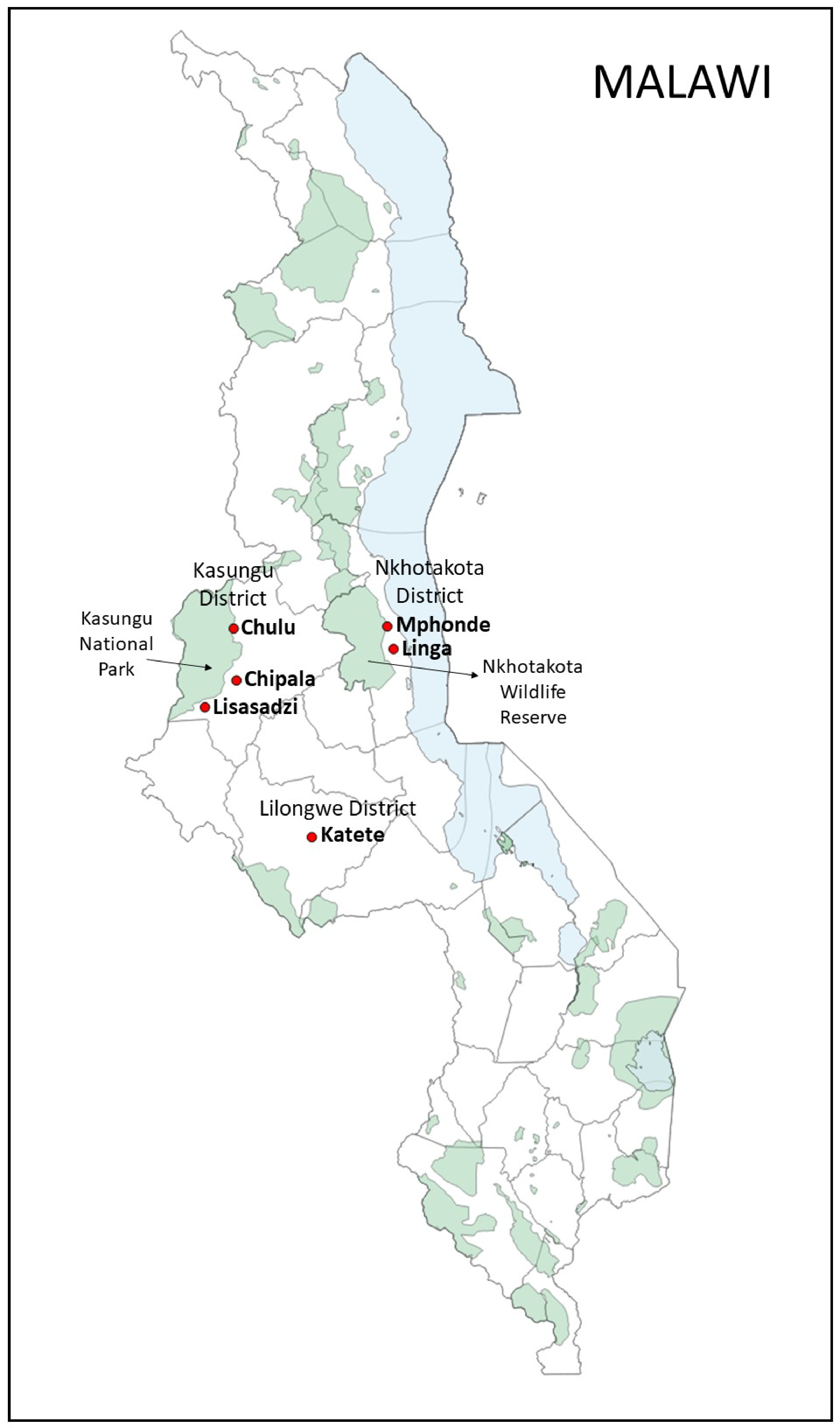

Supplement: Supplementary Figure — Workflow for molecular detection of African trypanosomes. The workflow depicts structural steps for detection of African animal and human trypanosomes with serial arrows to the right for cattle samples, and a single downward arrow for human samples. The section on the identification of trypanosome species by sequencing is divided into three steps: library preparation, sequencing and basecalling, and de-multiplexing. Bioinformatic analyses required in the experiments are framed in red borderline. [file parasite-27-46-s4.zip › R3_Fig 1. Malawi Map_300dpi_png.png]

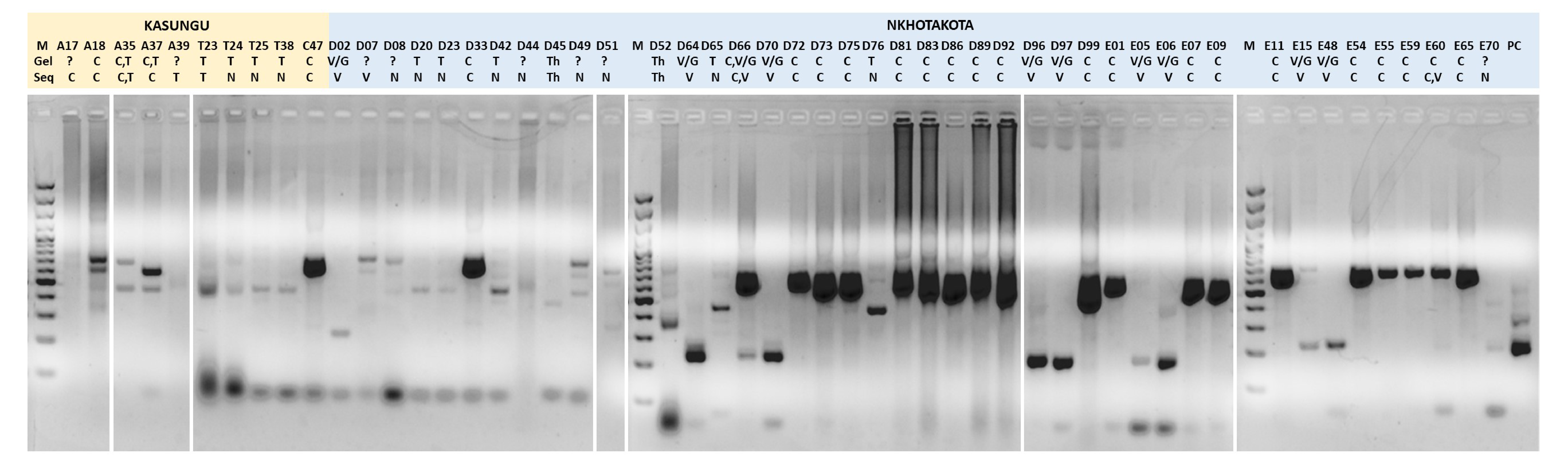

Supplement: Supplementary Figure — Workflow for molecular detection of African trypanosomes. The workflow depicts structural steps for detection of African animal and human trypanosomes with serial arrows to the right for cattle samples, and a single downward arrow for human samples. The section on the identification of trypanosome species by sequencing is divided into three steps: library preparation, sequencing and basecalling, and de-multiplexing. Bioinformatic analyses required in the experiments are framed in red borderline. [file parasite-27-46-s4.zip › R3_Fig 2. Gel image_300dpi_png.png]

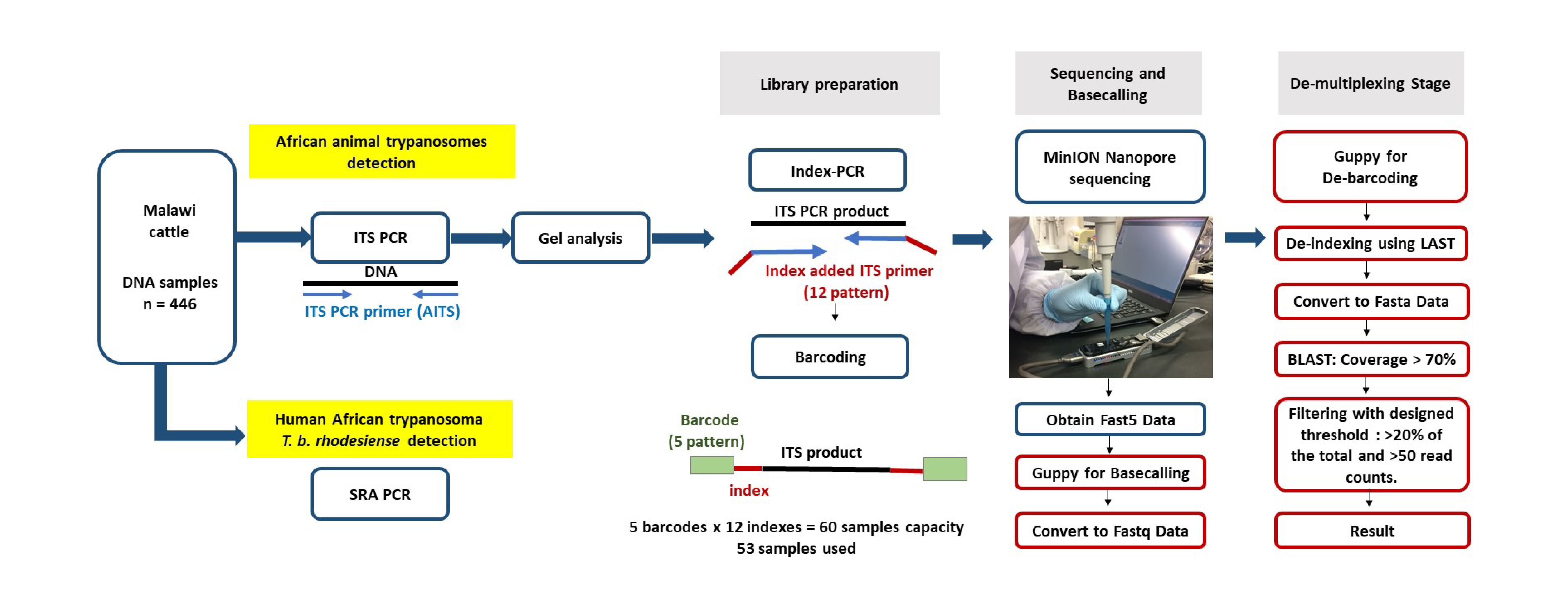

Supplement: Supplementary Figure — Workflow for molecular detection of African trypanosomes. The workflow depicts structural steps for detection of African animal and human trypanosomes with serial arrows to the right for cattle samples, and a single downward arrow for human samples. The section on the identification of trypanosome species by sequencing is divided into three steps: library preparation, sequencing and basecalling, and de-multiplexing. Bioinformatic analyses required in the experiments are framed in red borderline. [file parasite-27-46-s4.zip › R3_Supp. Figure_300dpi_png.png]
